# Maternal BMI and diabetes in pregnancy: Investigating variations between ethnic groups using routine maternity data from London, UK

**DOI:** 10.1371/journal.pone.0179332

**Published:** 2017-06-22

**Authors:** Erin Nishikawa, Laura Oakley, Paul T. Seed, Pat Doyle, Eugene Oteng-Ntim

**Affiliations:** 1 Faculty of Epidemiology and Population Health, London School of Hygiene and Tropical Medicine, London, United Kingdom; 2 Women's Health Academic Centre, King’s College London, London, United Kingdom; 3 Department of Women's Health, Guy’s and St Thomas’ NHS Foundation Trust, London, United Kingdom; University of Missouri Columbia, UNITED STATES

## Abstract

**Objective:**

To investigate the ethnicity-specific association between body mass index (BMI) and diabetes in pregnancy, with a focus on the appropriateness of using BMI cut-offs to identify pregnant women at risk of diabetes.

**Study design:**

Analysis of routinely-collected data from a maternity unit in London, UK. Data were available on 53 264 women delivering between 2004 and 2012. Logistic regression was used to explore the association between diabetes in pregnancy and BMI among women of different ethnicities, and adjusted probability estimates were used to derive risk equivalent cut-offs. ROC curve analysis was used to assess the performance of BMI as a predictor of diabetes in pregnancy.

**Results:**

The prevalence of diabetes in pregnancy was 2.3% overall; highest in South and East Asian women (4.6% and 3.7%). In adjusted analysis, BMI category was strongly associated with diabetes in all ethnic groups. Modelled as a continuous variable with a quadratic term, BMI was an acceptable predictor of diabetes according to ROC curve analysis. Applying a BMI cut-off of 30 kg/m^2^ would identify just over half of Black women with diabetes in pregnancy, a third of White (32%) and South Asian (35%) women, but only 13% of East Asian women. The ‘risk equivalent’ (comparable to 30 kg/m^2^ in White women) threshold for South Asian and East Asian women was approximately 21 kg/m^2^, and 27.5 kg/m^2^ for Black women.

**Conclusions:**

This study suggests that current BMI thresholds are likely to be ineffective for diabetes screening in South and East Asian women, as many cases of diabetes will occur at low BMI levels. Our results suggest that East Asian women appear to face a similarly high risk of diabetes to South Asian women. Current UK guidelines recommend diabetes screening should be offered to all pregnant South Asian women; extending this recommendation to include women of East Asian ethnicity may be appropriate.

## Introduction

Gestational diabetes mellitus (GDM) is defined as glucose intolerance that is first diagnosed during pregnancy[1]. Pregnancies complicated by diabetes are associated with an increased risk of negative outcomes for both the mother and child[2–4]. Additionally, as 40–60% of women with GDM go on to develop type 2 diabetes in later life, prevention and treatment of this disease has both short and long term implications[2, 5].

It is widely accepted that obesity is a risk factor for GDM. A recent meta-analysis found that the risk of an obese pregnant woman developing GDM was four times higher than that of a normal-weight woman[2]. In the United Kingdom, the National Institute for Health and Care Excellence (NICE) recommends that all women who are classified as obese should be screened for GDM in their pregnancy[1]. There has been concern about the definition of obesity as well as the usefulness of the standard BMI cut-offs in certain ethnic groups, particularly Asian populations[6]. In 2004, a World Health Organization (WHO) consultation concluded that the current BMI cut-offs were not appropriate in Asian populations, as they experienced a high risk for obesity-related diseases at a BMI lower than 25 kg/m^2^. Due to limited research evidence, no firm recommendations were made regarding alternative cut-offs, although the WHO did suggest further public health action points of 23.0 kg/m^2^, 27.5 kg/m^2^, 32.5 kg/m^2^ and 37.5 kg/m^2^ be used for Asian populations[7].

More recent research has further investigated these findings. In Canada, Retnakaran et al. found that an increasing BMI caused a much larger increase in insulin resistance and higher prevalence of GDM in pregnant South and East Asian women when compared to White women[8]. In the US, Shah et al. found that screening women with a BMI >25 kg/m^2^ identified only 24.9% of Asian women with GDM, as opposed to 46.2% of White women, and 76.8% of Black women. A lower cut-off of 21 kg/m^2^ still only identified 68.4% of Asians[9]. In the UK, Bryant et al. reported that Pakistani women had a higher prevalence of GDM, however, as the risk increased continuously, a lower BMI threshold (27.5 kg/m^2^ rather than 30 kg/m^2^) was no more effective at identifying women at risk{Bryant, 2014 #2}.

Although these studies provide evidence for differences in gestational diabetes risk based on ethnicity, there is a limited amount of relevant research, particularly in UK populations[10]. It is still unclear whether ethnicity-specific BMI cut-offs would be useful, and if so, what these cut-offs should be. NICE guidelines on diabetes in pregnancy suggest that “minority ethnic family origin with a high prevalence of diabetes” (defined elsewhere as South Asian, black Caribbean or Middle Eastern) should be an indicator for offering screening for GDM; however some non-White groups (e.g. East Asian) do not fall within this classification[1].

The aim of this study was to investigate the association between BMI and the risk of diabetes in pregnancy and how this varies by ethnic group in a diverse London population, with a focus on the use of existing and risk-equivalent cut-offs.

## Material and methods

### Study design and setting

We included all women with singleton births at 22 weeks gestation and beyond delivering between 2004 and 2012 at a large maternity unit located in London, UK. Ethical approval was granted from the London School of Hygiene and Tropical Medicine Ethics Committee, and all identifiable patient information was removed from the dataset before analysis.

### Variable description

The primary outcome variable was maternal diabetes mellitus. The dataset did not distinguish between previous diabetes diagnosed before pregnancy and GDM diagnosed during pregnancy, so the variable was simply the presence or absence of diabetes mellitus in pregnancy.

The primary exposure variables were maternal BMI and ethnicity. BMI was calculated from the mother’s height and weight taken at the first booking appointment [weight in kg/(height in m)^2^], and classified according to the WHO BMI categories: underweight (<18.5 kg/m^2^), recommended weight (18.5–24.9 kg/m^2^), overweight (25.0–29.9 kg/m^2^), obese class I (30.0–34.9 kg/m^2^), obese class II (35.0–39.9 kg/m^2^), and obese class III (40+ kg/m^2^)(9). Implausible BMI values <14 and >60 were coded as missing. For investigating equivalent risk cut-offs, we modelled BMI as a continuous variable. We assessed potential non-linear effects of BMI using polynomial models.

Ethnicity was self-reported by women and recorded by a midwife at the first booking appointment. Five ethnicity categories were created: “White” (British, English, European, White-Other), “South Asian” (Asian, British-Asian, Asian-Other, Indian, Bangladeshi, Pakistani, Sri Lankan), “East Asian” (Chinese, Filipino, Japanese, Vietnamese), “Black” (Black, Black-British, Black-African, Black-Other, Caribbean, African), and “Other” (Latin American, Middle Eastern, Arabic, Mixed, Other).

Other covariates included maternal age at delivery (20 years, 20–24 years, 25–29 years, 30–34 years, 35–39 years, and 40+ years) and parity (nulliparous: no previous births, primiparous: one previous birth, and multiparous: two+ previous births). Deprivation was measured using the Index of Multiple Deprivation 2007, a recognized scoring system developed by the UK government that classifies areas on a relative measure of deprivation based on the mother’s postcode[11].

### Statistical analysis

Descriptive statistics were presented for all women and separately by ethnic group. Odds ratios for the association between diabetes and main explanatory variables (BMI and ethnicity) were calculated using univariate and multivariate logistic regression. Multivariate models were adjusted for other variables found to be independently associated with diabetes (p<0.05 using a Wald test), and not thought to be on the causal pathway. Interaction between BMI and ethnicity was investigated by including interaction terms in multivariate models. Robust standard errors were used to account for women who had more than one birth within the eight-year time period. We obtained adjusted probability estimates for diabetes by BMI stratified by ethnic group using the Stata ‘margins’ command following a model including BMI as a continuous variable. We used these predictions to estimate ‘risk equivalent’ BMI cutpoints using White women as the reference group. We performed ROC (receiver operating characteristic) curve analysis to assess the value of BMI as a predictor of diabetes among different ethnic groups. All analyses were performed using Stata 14 (Stata Corporation, College Station, Texas).

## Results

The study population included 53 264 mothers delivering singleton births between 2004 and 2012. Descriptive characteristics for the whole population are shown in Table 1. Roughly 60% of the women were of recommended BMI, 25% were overweight and 15% were obese. A small percentage were underweight (3.3%). Half the sample was of White ethnicity (50.4%). Over 30% of the women were aged 30–34, and almost 60% were nulliparous. The majority of women (almost 80%) fell within the two most deprived quintiles.

**Table 1:** Descriptive characteristics and univariate analysis of factors associated with diabetes in pregnancy.

Variable	Total Births		Diabetes*		Crude OR† (95%CI)	P-Value‡
	n	%	n	%		
**Maternal BMI**						
Underweight: <18.5	1397	3.28	10	0.72	0.53 (0.28–0.99)	0.048
Normal: 18.5–24.9	24,417	57.33	327	1.34	1.00	
Overweight: 25.0–29.9	10,495	24.64	340	3.24	2.47 (2.10–2.89)	<0.001
Obese: >30	6,282	14.75	485	7.72	6.16 (5.30–7.17)	<0.001
*Obese Class I*: *30*.*0–34*.*9*	*4*,*173*	*9*.*8*	*258*	*6*.*18*	4.85 (4.08–5.78)	<0.001
*Obese Class II*: *35*.*0–39*.*9*	*1*,*480*	*3*.*47*	*152*	*10*.*27*	8.43 (6.85–10.39)	<0.001
*Obese Class III*: *>40*.*0*	*629*	*1*.*48*	*75*	*11*.*92*	9.97 (7.56–13.16)	<0.001
*Total*	42,591	100	1162	2.73		
*Missing Data*	10,673	20.04	66			
**Obesity**§						
No	36,309	85.25	677	1.86	1.00	
Yes	6282	14.75	485	7.72	4.40 (3.88–5.00)	<0.001
Total	42,591	100	1,162	2.73		
Missing Data	10,673	20.04	66			
**Ethnicity**						
White	26,433	50.4	405	1.53	1.00	
S Asian	2957	5.64	135	4.57	3.07 (2.47–3.83)	<0.001
E Asian	1623	3.09	60	3.70	2.47 (1.86–3.27)	<0.001
Black	17,313	33.01	522	3.02	2.00 (1.73–2.30)	<0.001
Other	4119	7.86	91	2.21	1.45 (1.14–1.85)	0.002
*Total*	52445	100	1213	2.31		
*Missing Data*	819	1.54	15			
**Maternal Age**						
<20	2039	3.83	10	0.49	0.27 (0.14–0.51)	<0.001
20–24	7215	13.55	85	1.18	0.65 (0.50–0.85)	0.002
25–29	12,121	22.75	218	1.80	1.00	
30–34	17,461	32.78	412	2.36	1.32 (1.12–1.56)	0.001
35–39	11,436	21.47	359	3.14	1.77 (1.48–2.11)	<0.001
40+	2,992	5.62	144	4.81	2.76 (2.22–3.43)	<0.001
*Total*	53264	100	1228	2.31		
*Missing Data*	0	0	0			
**Parity**						
Nulliparous	30,843	57.98	527	1.71	1.00	
Parity 1	13,185	24.79	355	2.69	1.59 (1.40–1.81)	<0.001
Parity 2+	9165	17.23	345	3.76	2.25 (1.95–2.59	<0.001
*Total*	53,193	100	1227	2.31		
*Missing Data*	71	0.13	1			
**Index of Deprivation**						
1 (least deprived)	1635	3.09	38	2.32	0.82 (0.56–1.19)	0.3
2	3056	5.78	53	1.73	0.61 (0.53–0.82)	0.002
3	6070	11.48	114	1.88	0.66 (0.53–0.82)	<0.001
4	23,983	45.35	502	2.09	0.74 (0.64–0.84)	<0.001
5 (most deprived)	18,135	34.30	512	2.82	1.00	
*Total*	52879	100	1219	2.31		
*Missing Data*	385	0.71	9			

*Diabetes in pregnancy

^†^OR calculated using univariate logistic regression

^‡^P-values calculated using Wald tests

^§^BMI> 30 kg/m^2^

**Fig 1:**
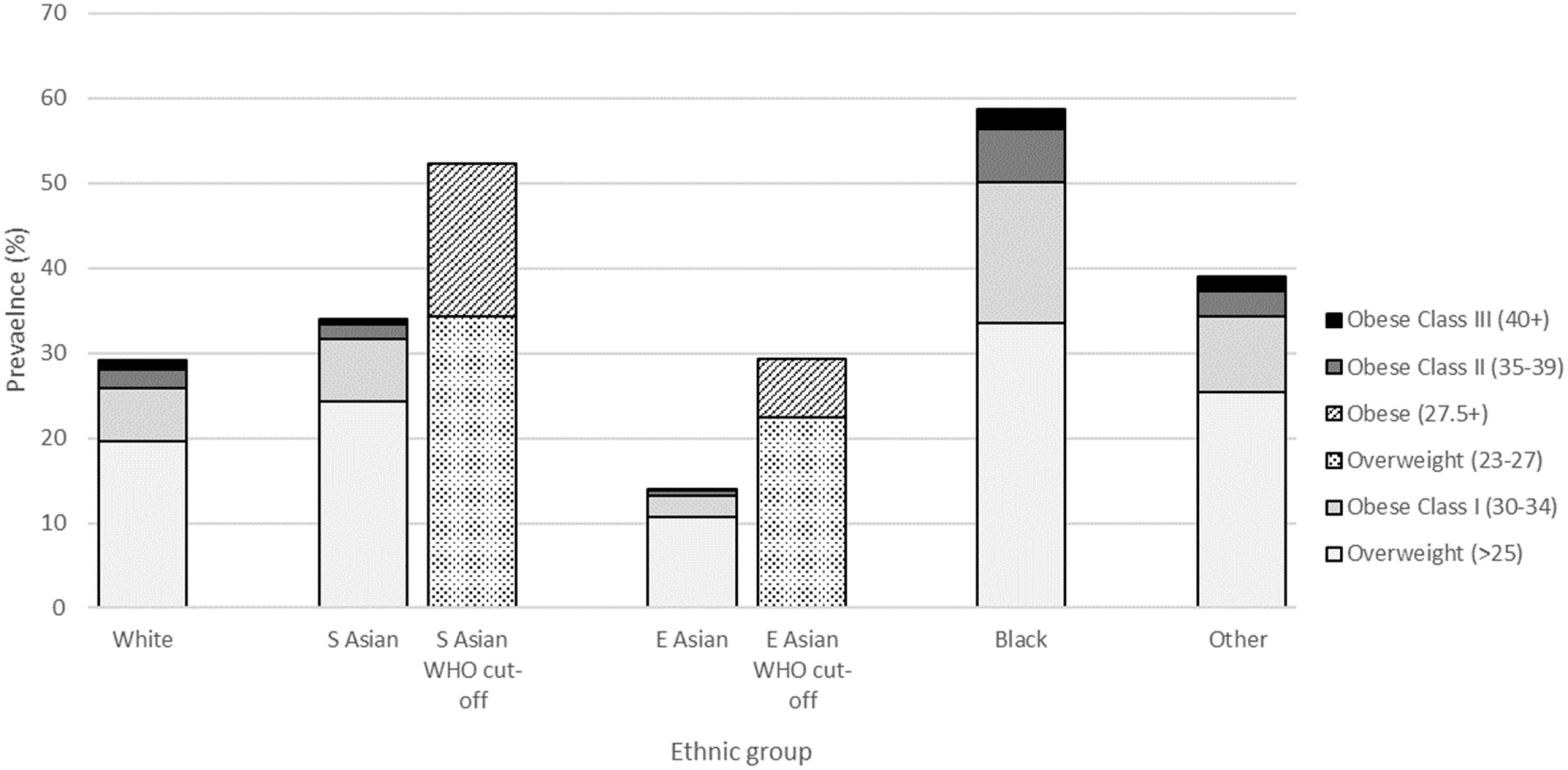
Prevalence of overweight/obesity by ethnic group, using conventional and Asian-specific thresholds.

Differences in BMI distributions by ethnic group are shown in Fig 1. Using conventional BMI thresholds, Black women had the highest proportion of both overweight (34%) and obesity (25%). The next highest level of overweight and obesity was in women of other ethnicities (25% overweight, 13.5% obesity), followed by South Asian (24.3% overweight, 9.7% obesity) and White women (19.6%, 9.5%). East Asian women had the lowest proportions of both overweight and obesity (10.8%, 3.3%). Applying Asian-specific BMI cut-offs (overweight 23–27.4 kg/m^2^, obesity ≥27.5 kg/m^2^) in this population resulted in 52.3% of South Asian women classified as overweight or obese, compared to 34.0% with conventional thresholds. The percentage of East Asian women classified as overweight or obese doubled with the use of Asian-specific BMI cut-offs (29.2% vs. 14.0%).

The prevalence of diabetes in the total population was 2.3%. South Asians had the highest prevalence of diabetes at 4.6%, and White women had the lowest prevalence at 1.5% (Table 1).

In univariate analysis the odds of diabetes increased with increasing BMI category, and all non-White ethnicities were associated with a higher odds of diabetes (Table 1). The odds of diabetes increased both with increasing maternal age, and increased parity. Compared to women living in the most deprived quintile, women living in less deprived quintiles had lower odds of diabetes.

Odds ratios adjusted for maternal age, parity and deprivation are presented for the main explanatory variables BMI and ethnicity are presented in Table 2. BMI remained associated with diabetes after adjustment for other factors. Overweight women experienced 2.37 higher odds, and obese women, 5.88 higher odds of diabetes than that of normal weight women (95% CI 2.00–2.80, p<0.001; and 4.97–6.96, p<0.001 respectively). This trend continued to obese class III women, where the adjusted odds ratio (aOR) reached 9.99 (95% CI 7.46–13.38, p<0.001). Using the traditional BMI cut-off of 30 kg/m^2^ for obesity, the adjusted odds of diabetes in obese women were 3.97 times that of non-obese women (95% CI 3.44–4.58, p<0.001). All non-White ethnicities continued to be strongly associated with a higher odds of diabetes in adjusted analysis. The highest odds were observed for South Asian and East Asian ethnicities (South Asian aOR 3.15, 95% CI 2.51–3.97; East Asian aOR 3.10, 95% 2.29–4.19).

**Table 2:** Multivariate analysis of factors associated with diabetes in pregnancy.

Variable	Adjusted OR* (95%CI)	P-value†
**Maternal BMI**		
Underweight: <18.5	0.53 (0.28–1.00)	0.051
Normal: 18.5–24.9	1.00	
Overweight: 25.0–29.9	2.37 (2.00–2.80)	<0.001
Obese: >30	5.88 (4.97–6.96)	<0.001
*Obese Class I*: *30*.*0–34*.*9*	4.64 (3.84–5.61)	<0.001
*Obese Class II*: *35*.*0–39*.*9*	8.08 (6.44–10.14)	<0.001
*Obese Class III*: *>40*.*0*	9.99 (7.46–13.38)	<0.001
**Obesity**‡		
No	1.00	
Yes	3.97 (3.44–4.58)	<0.001
**Ethnicity**		
White	1.00	
S Asian	3.15 (2.51–3.97)	<0.001
E Asian	3.10 (2.29–4.19)	<0.001
Black	1.28 (1.09–1.50)	0.003
Other	1.31 (1.02–1.70)	0.036

*AOR calculated using multivariate logistic regression, adjusted for maternal age, parity, deprivation and either BMI or ethnicity.

^†^P values calculated using Wald tests.

^‡^BMI ≥ 30 kg/m^2^

Table 3 displays the performance parameters of different BMI cut-offs for each ethnic group. A BMI cut-off of 25 kg/m^2^ would capture 59% of all White women with diabetes in pregnancy, 68% of South Asian women, 86% of Black women, but only 37% of East Asian women. Applying a BMI cut-off of 30 kg/m^2^ would identify just over half of Black women with diabetes in pregnancy, a third of White (32%) and South Asian (35%) women, but only 13% of East Asian women.

**Table 3:** Sensitivity, specific, PPV and NPV for specified BMI cut-offs, by ethnic group.

BMI cut-off	Ethnic group	sensitivity	specificity	PPV	NPV
**20 kg/m**^**2**^	*White*	95.2%	14.0%	2.1%	99.4%
	*South Asian*	95.0%	15.8%	5.9%	98.6%
	*East Asian*	88.9%	26.5%	4.8%	98.3%
	*Black*	98.8%	6.2%	3.8%	99.2%
**21.5 kg/m**^**2**^	*White*	87.5%	33.7%	2.4%	99.3%
	*South Asian*	93.6%	31.7%	7.0%	98.9%
	*East Asian*	74.1%	53.5%	6.2%	98.0%
	*Black*	96.8%	14.6%	4.0%	99.2%
**23 kg/m**^**2**^	*White*	75.8%	52.6%	2.9%	99.1%
	*South Asian*	85.7%	49.4%	8.5%	98.3%
	*East Asian*	61.1%	72.1%	8.4%	97.8%
	*Black*	92.7%	25.5%	4.4%	98.9%
**25 kg/m**^**2**^	*White*	59.2%	71.4%	3.8%	98.9%
	*South Asian*	67.7%	67.9%	10.4%	97.4%
	*East Asian*	37.0%	86.9%	10.6%	97.1%
	*Black*	86.2%	42.3%	5.3%	98.8%
**27.5 kg/m**^**2**^	*White*	43.9%	84.2%	5.0%	98.8%
	*South Asian*	47.6%	83.6%	13.8%	96.7%
	*East Asian*	25.9%	94.0%	15.2%	96.8%
	*Black*	72.1%	61.5%	6.5%	98.0%
**30 kg/m**^**2**^	*White*	32.1%	90.0%	6.3%	98.6%
	*South Asian*	34.7%	91.7%	18.7%	96.2%
	*East Asian*	13.0%	97.1%	15.9%	96.4%
	*Black*	56.3%	75.9%	8.0%	97.9%

PPV: Positive predictive value, NPV: Negative predictive value

**Fig 2:**
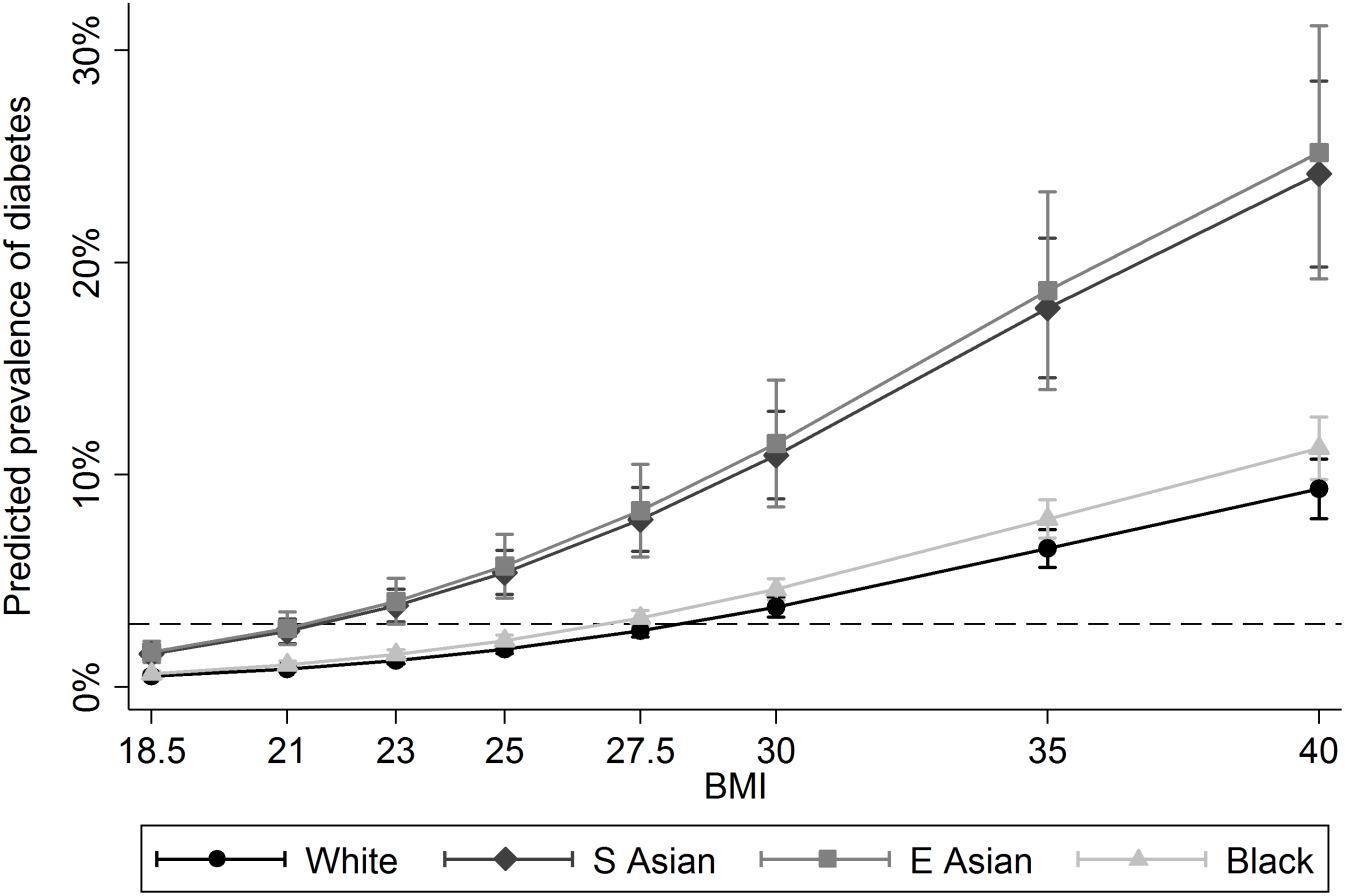
Predicted prevalence of diabetes by BMI and ethnic group.

The fit between BMI as a continuous predictor (in 1 kg/m^2^ increments) and risk of DM was slightly improved by using a quadratic polynomial term. Adjusted estimates of the prevalence of diabetes by BMI and ethnic group calculated using BMI as a quadratic term are presented in Fig 2. This figure shows an increased risk of diabetes at all BMI points for South Asian and East Asian women. At the conventional threshold of ‘overweight’ (BMI ≥25) and ‘obese’ (BMI ≥30), the prevalence of diabetes in White women was 1.7% and 3.0%. The corresponding figures for South Asian women were 5.1% and 9.0% with similar figures for East Asian women (5.0% and 8.5%). The dashed line on Fig 2 represents the prevalence of diabetes in White women at BMI 30. The ‘risk equivalent’ (comparable to 30 kg/m^2^ in White women) threshold for South Asian and East Asian women was approximately 21 kg/m^2^, and 27.5 kg/m^2^ for Black women.

**Fig 3:**
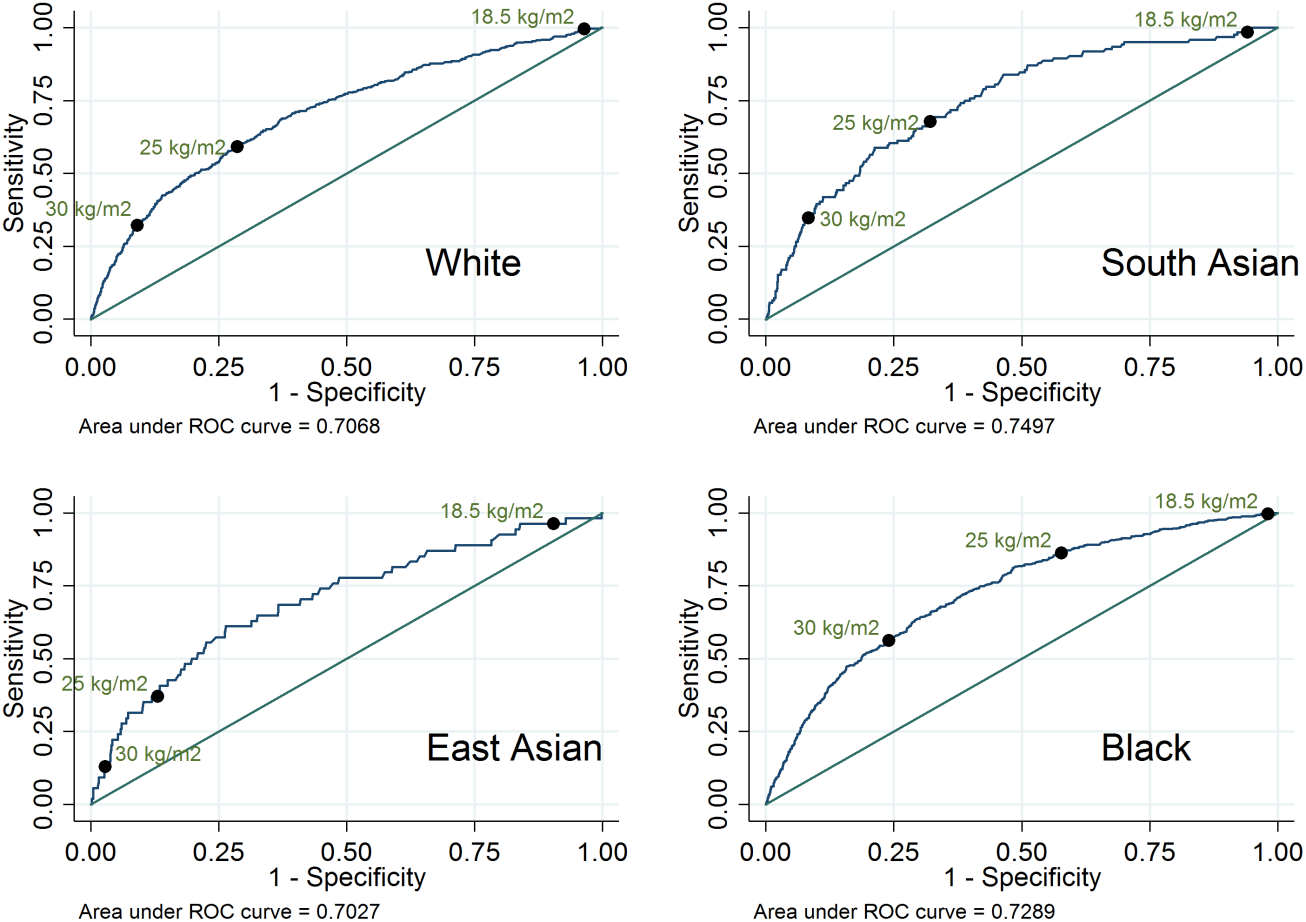
ROC curves for diabetes in pregnancy by BMI, by ethnic group.

Using BMI as a continuous predictor, the area under the ROC curve was 0.75 for South Asian women, 0.73 for Black women, 0.71 for White women, and 0.70 for East Asian women (Fig 3). These results suggest that BMI is an acceptable predictor of diabetes in pregnancy for all ethnic groups.

### Missing data

Data on BMI was missing for 20% of women. For all other variables, missing data was less than 2%. To assess any patterns, BMI was cross-tabulated with variables of interest (S1 Table). Women with missing BMI were less likely to have diabetes in pregnancy (0.6% vs 2.7%). They were slightly more likely to live in less deprived areas (4.8% vs 2.7% in the 1^st^ quintile), and to be nulliparous (63.9% vs 56.5%). Women with missing BMI were very slightly older (29.5% vs 26.5% aged ≥35) and slightly less likely to be East Asian (2.6% v 3.2%).

## Discussion

In this study conducted on a large, diverse London population, the prevalence of diabetes in pregnancy was 2.3%. This is consistent with national prevalence estimates by the Royal College of Obstetricians and Gynaecologists in 2011, which state that GDM affects up to 3.5% of all pregnancies[12]. We found that increasing BMI was associated with a higher odds of diabetes after adjusting for ethnicity, maternal age, parity and deprivation[1, 2, 5, 13–15]. Ethnicity was also associated with diabetes; after adjustment, South and East Asian women had three times higher odds of diabetes in pregnancy compared to White women. This finding is consistent with previous studies[8, 13, 16–19], many of which have also suggested that obesity affects GDM risk differently by ethnicity, with an increased risk in South and/or East Asian women specifically[8, 13, 14, 16, 17, 20]. We found some evidence that the association between obesity and diabetes risk varied by ethnicity when BMI was used as a categorical variable (p value for interaction 0.043), but there was little evidence of interaction when BMI was modelled as continuous with a quadratic term.

Our findings confirm that BMI is an acceptable predictor of diabetes in pregnancy. We found that both South and East Asian women were at a higher risk for diabetes at all specified BMI points when compared to other women. The predicted diabetes risk at all BMI points was similar for South and East Asians, despite a much lower prevalence of overweight and obesity in East Asians (10.8% and 3.3%) compared to South Asians (24.3% and 9.7%). This finding is of particular interest, as East Asian ethnicity is not generally considered a risk factor for gestational diabetes. A number of mechanisms have been proposed to explain why Asians have a higher risk of diabetes than people of European descent. One such explanation is that Asians have a genetic predisposition for insulin resistance due to mutations in various genes that code for proteins along the insulin pathway. Several genetic variants that have been isolated were found to be associated with type 2 diabetes in different Asian populations[21]. Research also suggests evidence for a thrifty gene, which allows individuals to store calories more efficiently in times of starvation. As Asians may have been more exposed to starvation, especially in developing countries such as those in South Asia, they may be more likely to inherit the gene. However, once exposed to the high-fat diets in the UK, this gene may make it more difficult to control body weight[14, 21, 22]. South Asians have also been found to have more central adiposity which is associated with increased insulin resistance[8, 14, 21, 22].

Current NICE guidelines specify a number of risk factors for diabetes in pregnancy, including minority ethnic family origin (including South Asian, but not East Asian), and obesity (defined as ≥30 kg/m^2^)[1]. There has been considerable debate about implementing lower cut-offs to define obesity among Asian groups, as suggested by the WHO. We suggest that this is not a solution: in our study, a cut-off of 27.5 kg/m^2^ corresponds to a predicted diabetes prevalence of 6.9% in South Asians and 6.6% in East Asians which is still considerably higher than the 3.0% predicted in White women at the same BMI level. NICE guidelines (2013) on the prevention of ill health in minority ethnic group populations made several research recommendations, including the need to assess what the BMI cut-off points should be among different ethnic groups in order to be “risk equivalent” for predicting obesity-related diseases[23]. Although this guideline was made for the general (non-pregnant) population, other studies have made similar calls for research in pregnant women[8, 10]. We attempted to derive risk equivalent cut-offs to determine the appropriate BMI threshold needed to detect a similar prevalence of diabetes among non-White women to that currently observed in White women at the conventional 25 kg/m^2^ and 30 kg/m^2^ cut-offs. To detect a diabetes prevalence of 3.0% in South and East Asian women (the prevalence detected in White women at BMI 30 kg/m^2^), the cut-off would need to be roughly 21 kg/m^2^. In this study population, 75% of South Asian women and 56% of East Asian women had a BMI ≥21 kg/m^2^. Applying ‘risk equivalent’ cut-offs to South and East Asian women would therefore result in the majority of women from these ethnic groups being classified as high risk due to BMI. A more effective method for identifying Asian women with GDM would be to implement universal screening for all Asian women regardless of their BMI. The inappropriateness of defining a BMI of ≥30 kg/m^2^ as a risk factor for diabetes in pregnancy among South and East Asian women is supported by a study conducted among Chinese, Malay and Indian women in Singapore, in which compared to universal screening, high risk screening (based on NICE guidelines) detected only one third of GDM cases in Chinese women and one half of cases in all women[24]. Shah et al. reached a similar conclusion in the US[9]. As GDM is one of several diseases associated with obesity, it is likely that a BMI cut-off of ≥30 kg/m^2^ is failing to identify women with other problems during pregnancy. Thus using the same BMI cut-offs for all women may increase inequalities between ethnic groups, as many Asian women are being wrongly assigned to low-risk care[10].

This study may have overestimated GDM prevalence, as there was no way of differentiating between previously diagnosed diabetes and true GDM. However, as gestational diabetes accounts for up to 90% of diabetes in pregnancy, it was considered an appropriate proxy[5] and the prevalence of diabetes in our study is consistent with estimates from other sources[1, 12]. As height and weight were measured at the first antenatal appointment, BMI values may be inaccurate for women who initiated antenatal care later in pregnancy. NHS statistics estimated that in 2012–13, 73% of women in England attended their first appointment within 12 weeks gestation[25]. Ethnicity was self-reported by the women and recorded by midwives; however, it is possible that ethnicity was assumed by midwives in some cases leading to possible misclassification. Collapsing ethnicity into five categories may also have been problematic. For example, Black women included women of Black African and Black Caribbean descent, who may experience different risks.

Despite the large number of births, diabetes in pregnancy was a rare outcome, which led to a small number of cases, especially when stratified by ethnic group. Missing data was another limitation, as 20% of women were missing information on BMI. Analysis revealed that few women with missing BMI data had diabetes. It may be that women who appeared to be of a healthy weight were assumed to have a normal BMI and not weighed. Since BMI was unlikely to be missing completely at random, this could have biased the estimates of diabetes prevalence in this study. Finally, although several important confounding factors were adjusted for, it is expected that residual confounding still exists. Other potential confounders include family history of diabetes, smoking, and hypertensive disorders.

The important strengths of this study were the large sample size and the ethnic diversity of the study population. A total of 53 264 women were included, and almost 50% were of non-White ethnicities. We were able to investigate risks for East Asians and South Asians separately, in contrast to many previous studies—particularly those conducted in North America—which combine these two together[9, 17]. Another important strength was the minimal selection bias, as this was a complete dataset including all deliveries occurring at the unit between 2004 and 2012. Furthermore, the completeness of the dataset meant that important confounders such as maternal age, parity and deprivation could be adjusted for.

## Conclusions

South and East Asian women are at higher risk of diabetes at considerably lower BMI thresholds compared to White and other non-White groups. Implementing the WHO recommended Asian-specific cut-off of 27.5 kg/m^2^ to define obesity has limited value, as at this BMI level South and East Asian women are likely to have a risk of diabetes over twice that of White women at 30 kg/m^2^. Any screening strategy which prioritises pregnant women for screening on the basis of BMI alone will not be an effective method for identifying women with gestational diabetes from South and East Asian backgrounds. Screening all Asian women for diabetes in pregnancy as a standard of care is likely to be a more effective method for identifying women at risk.

## Supporting information

S1 Table Comparison of characteristics between women with known BMI and women with missing BMI. (PDF)
