# Supplementary material for: Maternal BMI and diabetes in pregnancy: Investigating variations between ethnic groups using routine maternity data from London, UK
Source: PLoS One. 2017 Jun 22;12(6):e0179332. doi: 10.1371/journal.pone.0179332 (PMC5480876; doi:10.1371/journal.pone.0179332)
Supplement: S1 Table — (PDF) [file pone.0179332.s001.pdf]

Table S1. Comparison of characteristics between women with known BMI and women with missing BMI

Variable	Women with known BMI (n=42,590)		Women with missing BMI (n=10,674)		
	n	%	n	%	p value
**Diabetes**					<0.001
Yes	1162	2.7	66	0.6	
No	41428	97.3	10608	99.4	
**Ethnicity**					0.001
White	21,148	50.3	5,285	50.7	
S Asian	2,367	5.6	590	5.7	
E Asian	1,347	3.2	276	2.6	
Black	13,786	32.8	3,527	33.8	
Other	3,365	8.0	754	7.2	
Missing	577		242		
**Maternal Age**					<0.001
<20	1,603	3.8	436	4.1	
20-24	5,849	13.7	1,366	12.8	
25-29	9,794	23.0	2,327	21.8	
30-34	14,062	33.0	3,399	31.8	
35-39	8,952	21.0	2,484	23.3	
40+	2,330	5.5	662	6.2	
Mean age (SD)	31.5	(5.9)	31.8	(6.0)	
**Parity**					<0.001
Nulliparous	24,032	56.5	6811	63.9	
Parity 1	11,048	26.0	2137	20.0	
Parity 2+	7454	17.5	1711	16.1	
Missing	56		15		
**Deprivation**					<0.001
1 (least deprived)	1123	2.7	512	4.8	
2	2207	5.2	849	8.0	
3	4701	11.1	1369	13.0	
4	19,570	46.2	4413	41.8	
5 (most deprived)	14,720	34.8	3415	32.3	
Missing	269		116		
